# Supplementary material for: An open-source framework for large-scale, flexible evaluation of biomedical text mining systems
Source: J Biomed Discov Collab. 2008 Jan 29;3:1. doi: 10.1186/1747-5333-3-1 (PMC2276192; doi:10.1186/1747-5333-3-1)
Supplement: Additional file 1 — Evaluation platform source code. The additional file contains the software evaluation framework discussed in this paper. The most current release can be downloaded from SourceForge.net [1]. See the accompanying README file for instructions on installation and use. Also included in the distribution is a collection of UIMA wrappers for some commonly used BioNLP tools and annotated corpora. [file 1747-5333-3-1-S1.zip › bionlp_uima_repository-1.0.1/docs/api/edu/uchsc/ccp/uima/mention/CCPMention.html]

CCPMention (Center for Computational Pharmacology Util/UIMA API)


|  |  |  |
| --- | --- | --- |
| **Overview** \| **Package** \| **Class** \| **Tree** \| **Deprecated** \| **Index** \| **Help** | |  |
| **PREV CLASS**   **NEXT CLASS** | **FRAMES**    **NO FRAMES**     **All Classes** |  |
| SUMMARY: NESTED \| FIELD \| CONSTR \| METHOD | DETAIL: FIELD \| CONSTR \| METHOD |  |


---


## edu.uchsc.ccp.uima.mention Class CCPMention

```
java.lang.Object
  org.apache.uima.cas.impl.FeatureStructureImpl
      org.apache.uima.jcas.cas.TOP
          edu.uchsc.ccp.uima.mention.CCPMention
```

**All Implemented Interfaces:**: Cloneable, org.apache.uima.cas.FeatureStructure

**Direct Known Subclasses:**: CCPClassMention, CCPSlotMention

---

``` public class CCPMention extends org.apache.uima.jcas.cas.TOP ```

The superclass for all CCP Mentions (class mention, complex slot mention, and non-complex slot mention)
Updated by JCasGen Tue Aug 28 16:44:10 MDT 2007
XML source: /home/williamb/eclipse/RELEASED\_SOURCE\_CODE/descriptors/typeSystem/CCPTypeSystem.xml

---

| **Field Summary** | |
| --- | --- |
| `static int` | `type` |
| `static int` | `typeIndexID` |

**Fields inherited from class org.apache.uima.jcas.cas.TOP**

`addr, jcasType`


| **Constructor Summary** | |
| --- | --- |
| `protected` | `CCPMention()`             Never called. |
|  | `CCPMention(int addr, org.apache.uima.jcas.cas.TOP_Type type)`             Internal - constructor used by generator |
|  | `CCPMention(org.apache.uima.jcas.JCas jcas)` |


| **Method Summary** | |
| --- | --- |
| `String` | `getMentionName()`             getter for mentionName - gets The name of this mention. |
| `int` | `getTypeIndexID()` |
| `void` | `setMentionName(String v)`             setter for mentionName - sets The name of this mention. |

**Methods inherited from class org.apache.uima.jcas.cas.TOP**

`addToIndexes, addToIndexes, equals, getAddress, getCAS, getCASImpl, getLowLevelCas, hashCode, removeFromIndexes, removeFromIndexes`

**Methods inherited from class org.apache.uima.cas.impl.FeatureStructureImpl**

`clone, getBooleanValue, getByteValue, getDoubleValue, getFeatureValue, getFeatureValueAsString, getFloatValue, getIntValue, getLongValue, getShortValue, getStringValue, getType, prettyPrint, prettyPrint, prettyPrint, setBooleanValue, setByteValue, setDoubleValue, setFeatureValue, setFeatureValueFromString, setFloatValue, setIntValue, setLongValue, setShortValue, setStringValue, toString, toString`

**Methods inherited from class java.lang.Object**

`finalize, getClass, notify, notifyAll, wait, wait, wait`

**Field Detail**

### typeIndexID

```
public static final int typeIndexID
```

---


### type

```
public static final int type
```


**Constructor Detail**

### CCPMention

```
protected CCPMention()
```

:   Never called. Disable default constructor

---


### CCPMention

```
public CCPMention(int addr,
                  org.apache.uima.jcas.cas.TOP_Type type)
```

:   Internal - constructor used by generator

---


### CCPMention

```
public CCPMention(org.apache.uima.jcas.JCas jcas)
```


**Method Detail**

### getTypeIndexID

```
public int getTypeIndexID()
```

:   **Overrides:**: `getTypeIndexID` in class `org.apache.uima.jcas.cas.TOP`

---


### getMentionName

```
public String getMentionName()
```

:   getter for mentionName - gets The name of this mention.

---


### setMentionName

```
public void setMentionName(String v)
```

:   setter for mentionName - sets The name of this mention.


---


|  |  |  |
| --- | --- | --- |
| **Overview** \| **Package** \| **Class** \| **Tree** \| **Deprecated** \| **Index** \| **Help** | |  |
| **PREV CLASS**   **NEXT CLASS** | **FRAMES**    **NO FRAMES**     **All Classes** |  |
| SUMMARY: NESTED \| FIELD \| CONSTR \| METHOD | DETAIL: FIELD \| CONSTR \| METHOD |  |


---
